# Influence of dietary protein content on the chemico-physical profile of dry-cured hams produced by pigs of two breeds

**DOI:** 10.1038/s41598-019-55760-0

**Published:** 2019-12-13

**Authors:** Giuseppe Carcò, Stefano Schiavon, Ernestina Casiraghi, Silvia Grassi, Enrico Sturaro, Mirco Dalla Bona, Enrico Novelli, Luigi Gallo

**Affiliations:** 1 University of Padova, Department of Agronomy, Food, Natural Resources, Animals and Environment, Legnaro, PD 35020 Italy; 2 University of Milano, Department of Food, Environmental and Nutritional Sciences (DeFENS), Milano, 20133 Italy; 3 University of Padova, Department of Comparative Biomedicine and Food Science, Legnaro, PD 35020 Italy

**Keywords:** Animal physiology, Environmental impact

## Abstract

The use of low-protein (LP) feeds is a good strategy to reduce the environmental release of N compounds, but their influence on the quality of the products must be considered. This study explored the influence of LP diet and two pig breeds (BR) with different lean growth ability on the quality traits of dry-cured hams. We analysed 40 left dry-cured hams from pigs of two BR [Duroc-Danbred crosses (Danbred) and Duroc × Large White crosses (Anas)] fed either conventional (147 to 132 g/kg, crude protein) or LP diet. The LP had a crude protein content reduced by 20% with respect to the conventional. The differences in ham quality resulting from protein reduction were small, with a decrease of the protein and an increase of the lipid content of the ham slice in Anas, but not in Danbred (BR × Diet interaction; *P* = 0.043). Therefore, the use of LP would be feasible and sustainable, without detrimental effects on products. It was found the pig genotypes with different potentials for lean growth may affect the initial ham weight, fat cover and seasoning losses of hams, but they appear to affect little other chemical, physical and textural quality traits of the dry-cured hams.

## Introduction

Dry-cured ham is a traditional product in many Mediterranean areas^1^. At present, European Union quality control schemes recognise over 30 types of dry-cured ham, roughly half of which are classified Protected Designation of Origin (PDO) and half Protected Geographical Indication^2^.

It is generally agreed that ham quality depends on a combination of factors, including pig genotype, feeding and management practices, and curing procedures^3–5^. Producers of Italian hams with PDO, for example, must comply with specific requirements regarding pig genotype and feeding practices^6,7^. Previous studies on the effects of pig genotype and feed composition on the quality of raw hams^8,9^ have assumed the weight, back-fat cover and marbling of the raw material to be highly correlated with the final quality of the dry-cured hams^10,11^. When processing is standardised, it is reasonable to assume that the quality of the final product largely depends on the characteristics of the ham before curing^7^.

The use of low protein and low amino acid feeds (LP) has recently emerged as one of the best strategies to reduce the environmental release of N compounds from pig farms. In many experiments, LP diets have been found to increase fat cover thickness and intramuscular fat^12–14^. Greater fat cover thickness reduces the water losses during ripening, and this would exert positive effects on the final quality of the product^15,16^.

Heavy pig production in Italy has largely relied on the use of Large White, Landrace and Duroc breeds and their crosses, still considered as “traditional” genotypes. However, in the last decades is increasing the use of other breeds, and commercial hybrids, characterized by better farm performance and leaner carcasses^7^, such the Danish Duroc (Danbred) breed. Although a lean pig genotype is assumed to negatively affect the quality of dry-cured hams^6^, studies investigating the relationships between the genetic origin of pigs and the qualitative attributes of dry-cured hams are still lacking.

The aim of this study was to investigate the influence of LP diets on the characteristics of dry-cured hams obtained from two breeds of pigs with different lean growth potential.

## Results

### Weight changes

Diet and the BR × diet interaction had no influence on the weights and weight losses of the hams (*P* > 0.05, Table 1). Hams from barrows exhibited greater weight loss at salting (+34.2%, *P* = 0.047) but lower losses at deboning (−5.2%, *P* = 0.048) than those from gilts (data not in table). At the arrival at the ham factory, immediately after trimming, the trimmed Danbred hams had nearly 24% less fat cover thickness (*P* = 0.003) than the Anas hams, but were heavier both before salting (5.6%, *P* = 0.008) and after (5.7%, *P* = 0.004). However, they also had greater weight losses at seasoning (10.1%, *P* = 0.002) and deboning (9.3%, *P* = 0.001), so that, despite their greater initial weight, they had similar weights to the Anas hams at the end of seasoning and deboning (*P* = 0.08 and 0.29, respectively).

**Table 1:** Weights and losses of dry-cured hams obtained from pigs of different breeds (BR) and sex fed on conventional (CONV) or low protein (LP) diets.

	Diet (D)				Breed (BR)				D × BR
	CONV	LP	SEM	*P*	Anas	Danbred	SEM	*P*	*P*
Raw ham fat thickness^a^, mm	19.8	21.9	1.21	0.23	23.7	18.1	1.20	0.003	0.72
**Ham weight, kg**									
raw (trimmed)	14.7	14.8	0.21	0.70	14.4	15.2	0.21	0.008	0.36
after salting	14.4	14.4	0.18	0.92	14.0	14.8	0.18	0.004	0.29
after seasoning	10.1	10.1	0.15	0.72	9.92	10.3	0.15	0.08	0.37
after deboning	7.59	7.65	0.13	0.72	7.52	7.72	0.13	0.29	0.66
**Weight losses, kg**									
after salting^b^	0.37	0.46	0.04	0.15	0.39	0.43	0.04	0.48	0.97
after seasoning	4.65	4.69	0.10	0.77	4.44	4.89	0.10	0.002	0.55
after deboning^c^	7.13	7.18	0.13	0.78	6.84	7.47	0.13	0.001	0.29
**Weight losses, %**									
after salting	2.45	3.03	0.26	0.12	2.70	2.78	0.26	0.82	0.86
after seasoning	31.6	31.7	0.42	0.96	30.9	32.2	0.42	0.041	0.99
after deboning	48.4	48.4	0.49	0.93	47.6	49.2	0.49	0.030	0.56

^a^External fat cover thickness was measured with a ruler on the *Biceps femoris* muscle below the head of the femur of the fresh trimmed ham.

^b^Weight losses after salting were 0.47 kg for barrows and 0.35 kg for gilts (*P* = 0.047; SEM = 0.003).

^c^Weight losses after deboning were 6.97 kg for barrows and 7.33 kg for gilts (*P* = 0.048; SEM = 0.13).

### Chemical composition

**Figure 1:**
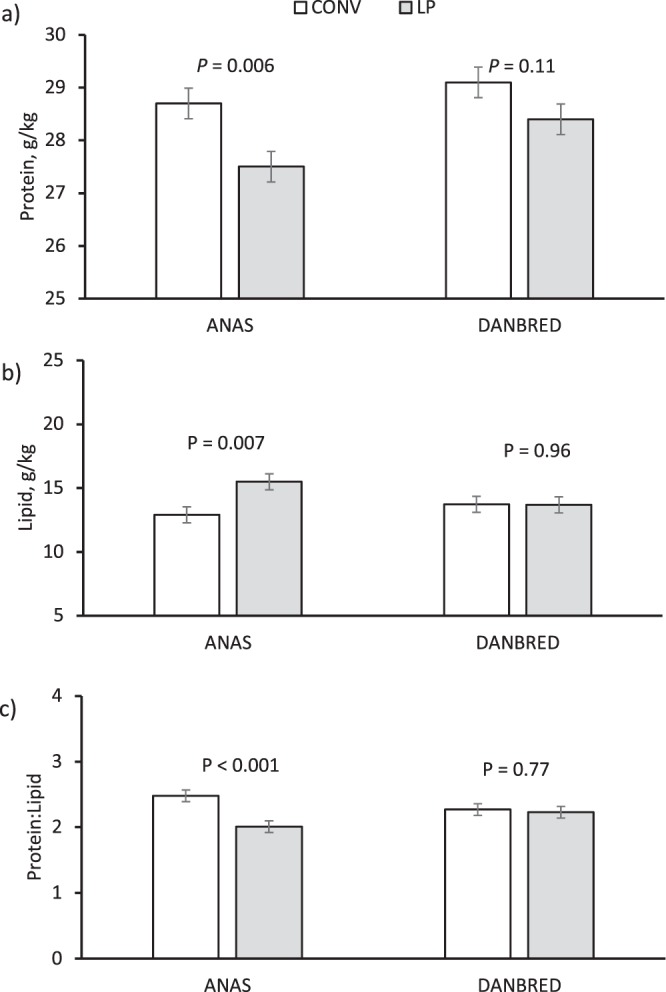
Influence of the genetic line × diet interaction on: (**a**) the lipid content (*P* = 0.045), and (**b**) the protein:lipid ratio (*P* = 0.023) of the dry-cured hams. Contrasts were run to evidence differences between conventional and low-protein diets within genetic line (n = 10, vertical bars indicate standard errors of the means).

**Figure 2:**
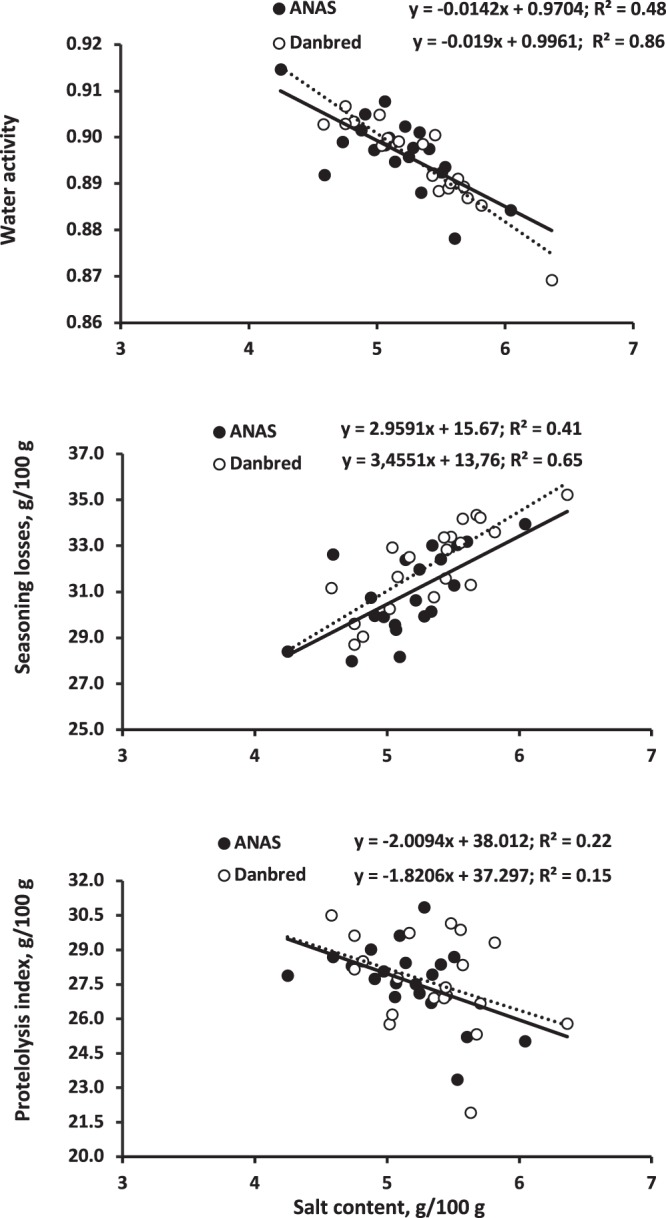
Relationships between salt content and (**a**) water activity in the lean part of the ham slice, (**b**) seasoning and deboning losses, and (**c**) proteolysis index.

**Figure 3:**
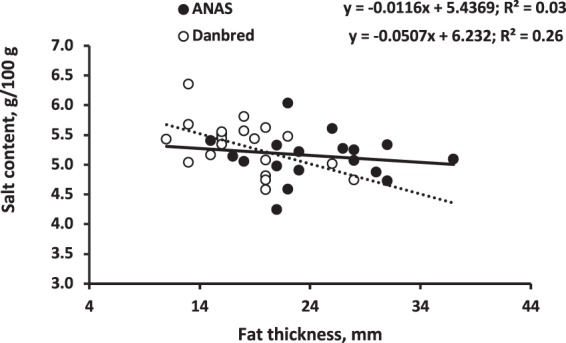
Relationship between the subcutaneous fat depth of the raw ham and the salt content of the lean part of the slice.

The hams from the Danbred pigs had a greater protein content (2.5%, *P* = 0.039) than those from Anas, and the hams from pigs fed the LP diet had a greater lipid content (+9.8%, *P* = 0.05) and a lower protein content (−3.1%, *P* = 0.003) and protein:lipid ratio (−10.5%, *P* = 0.008) than those from pigs fed conventional (CONV) diets (Table 2). However, diet interacted with BR for lipids (*P* = 0.045, Fig. 1), as the dietary protein reduction decreased the protein content (*P* = 0.006) and increased the lipid content (*P* = 0.07) and the protein:lipid ratio (*P* < 0.001) of the Anas hams compared with little variation (*P* > 0.05) in the Danbred hams. Breed and diet had no influence (*P* > 0.05) on the salt content of the lean part of the ham. The salt content was negatively correlated with water activity and the proteolysis index, and positively correlated with seasoning losses (Fig. 2), but there was only weak correlation between cover fat thickness of the fresh ham and the salt content (Fig. 3).

**Table 2:** Chemical characteristics of the dry-cured hams obtained from pigs of different breeds and sex^a^ fed on conventional (CONV) or low protein (LP) diets.

	Diet (D)				Breed (BR)				Tissue (T)		SEM	*P*	BR × D
	CONV	LP	SEM	*P*	Anas	Danbred	SEM	*P*	Slice, whole	Slice, lean part			*P*
**Chemical composition, g/kg**													
Water	507	505	3.3	0.67	508	504	3.3	0.39	481	531	3.2	<0.001	0.06
Protein^c^	289	280	2.1	0.003	281	288	2.1	0.039	278	291	1.7	<0.001	0.36
Ash	70.6	69.3	1.2	0.48	68.6	71.3	1.2	0.12	66.3	73.5	0.9	<0.001	0.49
Lipid^d^	133	146	4.5	0.05	142	137	4.5	0.44	174	105	4.2	<0.001	0.045^b^
Protein:Lipid^e^	2.37	2.12	0.06	0.008	2.25	2.25	0.06	0.99	1.67	2.82	0.06	<0.001	0.023^b^
Soluble protein^f^	81.5	78.9	1.0	0.07	80.3	80.2	1.0	0.98	—	—	—	—	0.54
Salt^f^	53.1	51.9	0.9	0.35	51.6	53.4	0.9	0.18	—	—	—	—	0.32
Proteolysis index^f^	0.277	0.275	0.004	0.68	0.276	0.276	0.004	0.92	—	—	—	—	0.69
***TBARS****,* **mg/kg**^**g**^													
*Biceps femoris* muscle	0.54	0.55	0.03	0.85	0.56	0.52	0.03	0.44	—	—	—	—	0.12
subcutaneous fat	0.56	0.48	0.03	0.07	0.52	0.53	0.03	0.77	—	—	—	—	0.11

^a^The fixed effect of sex was not significant.

^b^The least square means of the BR × D interaction are given in Fig. 2

^c^The *P* value of the BR × T interaction was 0.038 (Fig. 5).

^d^The *P* value of the BR × T interaction was 0.003 (Fig. 5).

^e^The *P* value of the BR** × **T interaction was 0.004 (Fig. 5).

^f^Measured on the lean part of the slice

^g^TBARS: thiobarbituric acid reactive substances.

**Figure 4:**
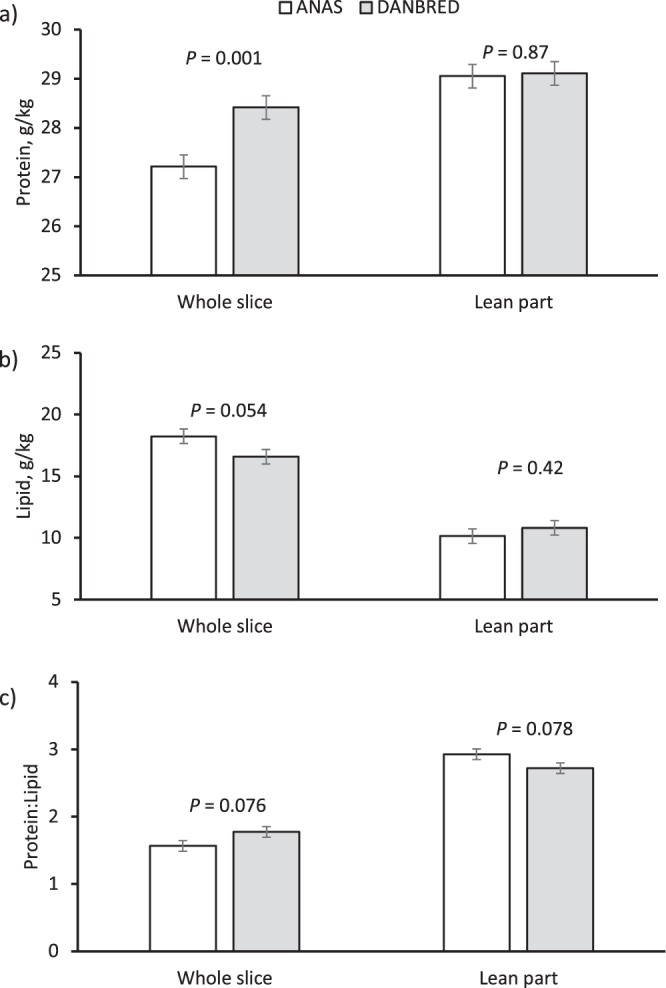
Influence of the genetic line × tissue interaction on: (**a**) the protein content (*P* = 0.038), (**b**) the lipid content (*P* = 0.003), and (**c**) the protein:lipid ratio (*P* = 0.004) of the whole and the lean part of the ham slice. Contrasts evidenced differences between GLs in the constituent contents of the whole and of the lean part of the slice (n = 10, vertical bars indicate SE of the means).

As expected, removing from the slice the subcutaneous fat increased water (10.4%, *P* < 0.001), protein (4.7%; *P < *0.001) and ash (10.9%, *P* < 0.001) content of the lean part of the slice and the protein:lipid ratio (68.9%, *P* < 0.001) and decreased lipid content (−39.7%, *P* < 0.001) with respect to the whole slice. However, a BR × tissue interaction was found for protein (*P* = 0.038) and lipid (*P* = 0.003) contents and for the protein:lipid ratio (*P* = 0.004). In fact, the whole slice from the Danbred hams had a 4.4% greater protein content (*P* = 0.001) and a 9.1% lower fat content (*P* = 0.054) than Anas hams, but the composition of the lean part was similar in the hams from the two breeds (*P* > 0.05, Fig. 4).

### Physical and textural traits

Diet and breed had little or no influence on pH, water activity, colour attributes and textural traits (*P* > 0.05, Table 3). However, almost all traits were significantly influenced by muscle (*P* < 0.001). Namely, the shear force was markedly greater in the *semimembranosus* muscle than in the *biceps femoris*. The hardness and chewiness values of the *biceps femoris* and the *quadriceps femoris* were almost twice those of the *semitendinosus* and *semimembranosus* muscles (*P* < 0.001). The *biceps femoris* had the greatest adhesiveness and the lowest cohesiveness.

**Table 3:** Physical characteristics of the different muscles of dry-cured hams obtained from pigs of different breeds and sex^1^ fed on conventional (CONV) or low protein (LP) diets.

	Diet (D)				Breed (BR)				Tissue (T)^2^						BR × D
	CONV	LP	SEM	*P*	Anas	Danbred	SEM	*P*	BF	QF	SM	ST	SEM	*P*	*P*
pH	5.49	5.52	0.01	0.07	5.51	5.50	0.01	0.39	5.51^bc^	5.53^c^	5.50^b^	5.48^a^	0.01	<0.001	0.49
Water activity^3^	0.90	0.90	0.002	0.76	0.90	0.89	0.002	0.36	—	—	—	—	—	—	0.84
**Colour:**															
Lightness (L*)	37.5	37.3	0.29	0.64	37.7	37.2	0.29	0.30	38.1^c^	35.6^b^	33.5^a^	42.7^d^	0.34	<0.001	0.37
Green-red (a*)	7.00	6.92	0.18	0.78	7.05	6.87	0.18	0.46	6.86^b^	8.28^d^	7.53^c^	5.18^a^	0.19	<0.001	0.77
Blue-yellow (b*)	8.24	8.30	0.12	0.75	8.26	8.28	0.12	0.92	7.47^a^	8.61^b^	7.02^a^	9.97^c^	0.14	<0.001	0.80
**Texture:**															
Shear force, N	32.8	33.6	1.10	0.59	33.0	33.4	1.10	0.81	21.4^a^	36.2^b^	42.5^c^	32.7^b^	1.56	<0.001	0.22
Hardness (30%), N	20.7	19.3	1.09	0.36	19.1	21.0	1.09	0.22	26.3^b^	24.0^b^	13.6^a^	16.2^a^	1.07	<0.001	0.30
Adhesiveness, N × s	−1.73	−1.73	0.08	1.00	−1.81	−1.66	0.08	0.20	−2.00^c^	−1.48^a^	−1.84^bc^	−1.62^ab^	0.09	<0.001	0.88
Cohesiveness	0.54	0.55	0.01	0.25	0.55	0.54	0.01	0.56	0.53^a^	0.60^b^	0.53^a^	0.52^a^	0.01	<0.001	0.51
Springiness	0.73	0.73	0.01	0.81	0.73	0.73	0.01	0.81	0.74	0.72	0.72	0.72	0.01	0.34	0.10
Chewiness, N	8.42	7.92	0.52	0.50	7.76	8.58	0.52	0.27	10.6^b^	10.6^b^	5.33^a^	6.18^a^	0.54	<0.001	0.65

^1^The fixed effect of sex was not significant.

^2^BF: *Biceps femoris*; QF: *Quadriceps femoris*; SM: *Semimembranosus muscle*; ST: *Semitendinosus muscle*.

^3^Measured on square samples (15 × 15 mm) taken from close to the *Biceps femoris* muscle.

### Fatty acid composition

The reduction in the dietary crude protein (CP) level had no significant influence on the FA composition of the ham tissues (*P* > 0.05, Table 4). The intramuscular and the subcutaneous FA profiles differed greatly, the former having a greater polyunsaturated FA content (*P* < 0.001), mainly due to the proportions of 18:2 *cis-*9, *cis-*12 (*P* < 0.001) and 20:4 cis-5, cis-8, cis-11, cis-14, and a lower monounsaturated FA content (*P* < 0.001), due to 18:1 *cis*−9 (*P* < 0.001). The FA profiles of the ham tissues were influenced by breed. The Danbred hams had a greater polyunsaturated FA content (*P < *0.001), mainly because of the 18:2 *cis*-9, *cis*-12 content (*P* < 0.001), and slightly lower amounts of some other monounsaturated and saturated FA compared with Anas hams.

**Table 4:** Fatty acid compositions of the intramuscular (IM) and subcutaneous (SC) fat of dry-cured hams obtained from pigs of different breeds (BR) and sex fed on conventional (CONV) or low protein (LP) diets.

	Diet (D)				Breeds (BR)				Tissue (T)				BR × D
	CONV	LP	SEM	*P*	Anas	Danbred	SEM	*P*	IM	SC	SEM	*P*	*P*
**Satured fatty acids (SFA) %**													
10:0	0.17	0.17	0.004	0.37	0.18	0.16	0.004	0.015	0.19	0.14	0.004	< 0.001	0.93
12:0^a^	0.13	0.12	0.005	0.24	0.13	0.12	0.003	0.010	0.12	0.12	0.003	0.020	0.35
14:0^b^	1.57	1.51	0.003	0.19	1.61	1.47	0.03	0.11	1.52	1.56	0.02	< 0.001	0.63
16:0	21.4	21.5	0.28	0.99	21.9	20.9	0.21	0.69	20.8	21.4	0.16	< 0.001	0.80
17:0	0.23	0.22	0.01	0.08	0.20	0.25	0.01	<0.001	0.23	0.22	0.01	0.63	0.06
18:0	8.79	9.09	0.12	0.06	8.96	8.88	0.12	0.64	9.18	8.66	0.12	0.003	0.009
20:0	0.10	0.10	0.003	0.62	0.11	0.10	0.003	0.012	0.11	0.10	0.003	0.007	0.08
Total SFA	30.8	33.1	0.71	0.19	32.6	32.1	0.72	0.52	32.5	32.2	0.44	0.52	0.69
**Monounsatured fatty acids (MUFA) %**													
16:1 cis-7^c^	0.39	0.38	0.01	0.43	0.35	0.42	0.01	<0.001	0.38	0.39	0.01	0.29	0.95
16:1 cis-9	2.96	2.78	0.07	0.09	2.86	2.87	0.07	0.88	3.05	2.69	0.05	<0.001	0.51
17:1 cis-10^d^	0.26	0.24	0.01	0.10	0.23	0.27	0.01	0.009	0.21	0.29	0.001	<0.001	0.54
18:1 cis-9	41.9	42.0	0.33	0.74	42.4	41.5	0.33	0.12	40.9	43.1	0.33	<0.001	0.82
18:1 cis-11	4.18	4.11	0.09	0.57	4.09	4.20	0.09	0.37	4.11	4.18	0.09	0.59	0.07
18:1 isomers^e^	0.32	0.31	0.01	0.60	0.32	0.32	0.01	0.96	0.28	0.35	0.01	<0.001	0.44
20:1 trans-11^f^	0.80	0.76	0.03	0.46	0.81	0.76	0.03	0.39	0.70	0.87	0.03	0.001	0.38
Total MUFA	51.0	50.8	0.33	0.75	51.4	50.4	0.33	0.12	49.9	52.0	0.34	<0.001	0.53
**Polyunsatured fatty acids (PUFA) %**													
18:2 cis-9, cis-12	13.8	13.5	0.19	0.33	13.1	14.2	0.19	<0.001	14.0	13.2	0.16	<0.001	0.83
18:2 trans-9, trans-12	0.14	0.14	0.02	0.90	0.13	0.16	0.02	0.21	0.22	0.06	0.02	<0.001	0.74
18:2 other isomers^g^	0.18	0.17	0.01	0.15	0.18	0.17	0.01	0.75	0.22	0.14	0.01	<0.001	0.21
18:3 cis-9, cis-12, cis-15	0.69	0.67	0.01	0.14	0.64	0.72	0.01	<0.001	0.65	0.71	0.01	<0.001	0.79
CLA sum	0.12	0.12	0.004	0.43	0.11	0.13	0.005	0.028	0.13	0.11	0.01	<0.001	0.26
20:2 cis-11, cis-14	0.65	0.62	0.02	0.41	0.61	0.66	0.02	0.13	0.59	0.68	0.004	<0.001	0.23
20:3 cis-8, cis-11, cis-14	0.18	0.17	0.01	0.32	0.18	0.17	0.01	0.95	0.24	0.11	0.02	<0.001	0.34
20:3 cis-11, cis-14, cis-17	0.14	0.12	0.01	0.07	0.11	0.13	0.01	0.64	0.13	0.13	0.01	0.77	0.92
20:4 cis-5, cis-8, cis-11, cis-14^h^	0.78	0.76	0.02	0.64	0.76	0.78	0.02	0.53	1.36	0.18	0.02	<0.001	0.51
Total PUFA	16.8	16.4	0.22	0.21	15.9	17.2	0.22	<0.001	17.7	15.4	0.18	<0.001	0.95
*n-3* fatty acids^i^	1.54	1.49	0.03	0.21	1.47	1.55	0.03	0.09	2.08	0.95	0.03	<0.001	0.93
*n-6* fatty acids	15.0	14.7	0.16	0.25	14.2	15.5	0.20	<0.001	15.4	14.4	0.16	<0.001	0.97
*n-6/n-3*	11.1	11.5	0.14	0.14	11.1	11.5	0.25	0.10	7.46	15.1	0.14	<0.001	0.78
Minor fatty acids ^j^	0.86	0.78	0.03	0.09	0.80	0.84	0.03	0.37	0.85	0.79	0.03	0.047	0.64

^a^C12:0 was 0.12 for barrows and 0.13 for gilts (*P* = 0.035; SEM = 0.03).

^b^The *P* value of the BR × T interaction was 0.019.

^c^C16:1 cis-7 was 0.37 for barrows and 0.40 for gilts (*P* < 0.001; SEM = 0.008).

^d^The *P* value of the D × T interaction was 0.032.

^e^The *P* value of the D × T interaction was 0.033.

^f^C20:1 trans-11 was 0.72 for barrows and 0.85 for gilts (*P* = 0.014; SEM = 0.03).

^g^The *P* value of the D × T interaction was 0.013.

^h^C20:4 cis-5, cis-11, cis-14 were 0.83 for barrows and 0.72 for gilts (*P* = 0.002; SEM = 0.02).

^i^*n-3* fatty acids were 1.56 for barrows and 1.47 for gilts (*P* = 0.002; SEM = 0.03).

^j^Minor fatty acids include: C6:0; C8:0; C10:1 *cis*-9; C11:0; C13:0; C14:1 *cis*-9; C15:0; C15:1 *cis*-10; C16:0 iso; C16:0 anteiso; C17:0 iso; C17:0 anteiso; C18:0 iso; C18:0 anteiso; C18:3 *cis*-6*, cis*-9*, cis*-12; C19:0; C21:0; C20:5 *n-3*; C22:0; C22:1 *trans*-13; C22:2 cis-13, cis-16, C23:0, C24:0; C24:1 *cis-*15, C22:6 *cis*-4*, cis*-7*, cis*-10*, cis*-13*, cis*-16*, cis*-19.

## Discussion

Feed characteristics and breeds are considered important sources of variation of the quality of dry-cured hams^4,6^. Most of the literature in this area has focussed on the influence of genetics and feeding on the quality of the raw ham^8,11,15^, whereas their effects on the characteristics of the dry-cured ham have been less investigated^17,18^. Furthermore, few experiments have compared the quality traits of dry-cured hams obtained from pigs of different breeds and fed on different diets under the same rearing conditions^6^.

The use of LP diets is an effective strategy for reducing the environmental release of N and its potential polluting effects^19^. Previous experiments in the Italian heavy pig industry have found that diets formulated to achieve a reduction from 146 to 117 g/kg of CP and from 7.3 to 5.8 g/kg of total lysine in early finishing (90 to 130 kg BW) and 133 to 108 g/kg of CP and 5.7 to 4.7 g/kg of total lysine in late finishing (130 to 165 kg BW) had negligible influence on growth performance^20^, weight of carcass and primary cuts, and yields of the dressed hams^21^, but greatly reduced N excretion^20^. Interestingly, Gallo *et al*.^15^ found that reducing the dietary CP content from 14 to 11% increased the subcutaneous fat thickness, decreased linoleic and polyunsaturated fatty acid in fat depots, and reduced seasoning losses in fresh hams destined for PDO dry-cured ham production. Similarly, Schiavon *et al*.^9^ found that the reduction in CP had little impact on raw ham characteristics, except for an increasing in ham subcutaneous fat covering and marbling scores.

The current experiment suggests that a LP diet has little overall influence on the chemical and physical profile of dry-cured ham, although the protein content and the protein: lipid ratio of the whole slice were lowered, and there was a tendency towards an increase in the lipid content with respect to the conventional diet. Previous work has already reported that feeding pigs with LP diets increases the proportion of fat in the carcass and in the meat^22,23^. These increases would depend on the replacement in the diets of some protein source with carbohydrates, which are more easily converted to fat^24^. However, the results of current experiment suggest that the response to LP diets would be at least partially dependent by the genetic background of the pig, as the Danbred pigs were less responsive of Anas one to the reduction of the dietary CP level.

Dietary CP reduction also tended to reduce the soluble protein content, but not the proteolysis index, and the values of the TBARS measured on the subcutaneous fat. The effect of these differences on the eating quality of the ham is unknown, and major research is required to clarify this. In any case, and in agreement with^25^, the differences in ham quality resulting from the use of LP diets seem very small, thereby confirming them as a valid strategy for sustainable production of dry-cured ham.

Pigs with different genetic backgrounds differ in growth rates, carcass composition, lean/fat ratios and adipose tissues characteristics^4^. As discussed in a companion paper^9^, the Danbred pigs used in the current experiment and fed restrictively exhibited greater feed efficiency (gain:feed, 0.271 *vs*. 0.269) and total carcass lean cuts (54.4 *vs*. 52.1 kg/100 kg carcass), and thinner carcass back-fat cover (30.2 *vs*. 34.1 mm) than the Anas pigs, but had the same average daily gain (0.703 *vs*. 0.700 kg/d). Furthermore, the fresh hams obtained from the Danbred pigs were 6% heavier with 22% less fat cover thickness and a 42% greater marbling score than the Anas pigs. These data show that when kept under the same feeding and rearing conditions the two breeds have different levels of leanness. The results were consistent with previous studies in which Danbred pigs were compared with the traditional genotypes used to produce Italian dry-cured hams^8,18^. Vitale *et al*.^18^ found that the thighs from Danbred were heavier but had lower fat thickness and seasoning aptitude, with losses greater than 30%, compared with other traditional breeds or commercial lines.

It is generally agreed that raw hams from lean pig genotypes are less suitable for the production of dry-cured hams because leaner carcasses and thinner subcutaneous fat cover are frequently associated with high seasoning losses, high salt absorption, increased dehydration and hardening of the meat, and the development of a salty flavour^26^. For these reasons, the consortia for the protection of PDO dry-cured hams restrict the breeds that can be used as boar line in the crossbreeding schemes aimed to originate heavy pigs for traditional dry-cured ham production. Italian Large White, Italian Landrace and Italian Duroc boars are always compliant with PDO dry-cured ham production, while several other breeds or genetic lines can be used as sires only if they originate from selection schemes having purposes consistent with those of this type of production^6^.

High fat cover and high intramuscular fat content of the ham are a barrier to water and salt penetration^4,27^. Seasoning losses in ham are known to be inversely related to the depth of fat cover^10^, which, in turn, is related to the depth of back-fat at the loin^28^. Rapid desiccation can also cause a crust to form on the surface, and once this has occurred, further diffusion of water is difficult so that the inner part of the ham becomes soft^1^. In the current experiment, and in line with expectations, seasoning losses were 4% higher in the Danbred than in the Anas hams, reflecting the leaner characteristics of the former breed. Despite the initial weight differences, at the end of seasoning the ham weight of the two breeds did not differ. In addition, at the end of seasoning, the lipid and the protein content of the ham lean part did not differ in the two breeds, but the Danbred ham still tended to be 5% richer in protein and 10% poorer in lipid than that from the Anas pigs, reflecting the different fat cover of the hams produced by the two breeds. This result was consistent with the values of fat thickness measured on the fresh hams. The fatty acid profiles of the various fatty depots in the ham showed there to be an 8% greater proportion of polyunsaturated fatty acid in the Danbred than in the Anas hams, consistent with the observation that a reduction in back-fat thickness is associated with an increase in the proportion of polyunsaturated fatty acid^8^.

Salt confers a salty flavour to the meat and diminishes the health properties of the ham^29,30^. Moreover, it plays an important role, in conjunction with lower water activity, in microbial inhibition^31^. The negative relationship between meat salt content and the proteolysis index found in the current experiment confirmed the anti-proteolytic properties of salt^32^. The salt content of the lean was also negatively related to water activity and positively related to seasoning losses.

Surprisingly, despite notable differences in fresh ham weight, the quantity and quality of fat, and seasoning losses, there were only small differences between the hams of the two breeds in other quality traits, such as salt content, soluble protein, proteolysis index, TBARS measured in the muscle and the adipose tissue, and physical and texture characteristics. The relationships between ham fat thickness and these quality traits, including the salt content, were small. The small correlation between fresh ham fat thickness, salt content and the other quality traits may be partly due to the small number of hams examined in the current experiment and the high degree of variation in some variables. Further experiments with a greater number of dry-cured hams are, therefore, needed.

In conclusion, there is potential to use LP diets in the Italian PDO dry-cured ham production as it reduces the N release into the environment but has little influence on several chemical and physical attributes of the hams. This study also provides evidence that hams originated from pigs of breeds characterized by different potentials for lean growth may differ for raw ham fat cover and seasoning losses, whereas differences in several specific chemical, physical and textural attributes of the dry-cured hams are less evident. Further researches are needed to investigate the influence of these factors on the sensory and eating properties of dry-cured hams.

## Methods

### Ethics statement

All experimental procedures were reviewed and approved (# 29562/2012) by the Ethical Committee for the Care and Use of Experimental Animals (CEASA) of the University of Padua, Italy. All the procedures and methods were completed in agreement with the Guideline for the Care and Use of Agricultural Animals in Agricultural Research and Teaching^33^.

### Dry-cured hams origin and experimental design

This study used 40 dry-cured hams originated from a previous feeding trial, and details of the animals, diets, growth performances, and raw ham characteristics can be found in that paper^9^. Briefly, the feeding trial involved 96 pigs of four breeds fed restrictively CONV or LP feeds from 89 to 165 kg BW, according to the rules of PDO dry-cured ham production^6,34^. The CONV feeds contained 147 and 132 g/kg of CP and 6.0 and 4.4 g/kg of standardized ileal digestible (SID) lysine (Lys) in the early (89–120 kg BW) and late (121–165 kg BW) finishing periods, respectively. The LP diets contained only 119 and 103 g/kg of CP and 4.8 and 3.5 g/kg of SID Lys in the early and late finishing periods, respectively, whereas the energy content was the same in the two feeding programs. After slaughtering and carcass dissection, all hams were sent to “Testa & Molinaro” ham factory [San Daniele del Friuli (UD), Italy] to be processed into dry-cured hams in accordance with San Daniele procedures^34^. From these, 40 left dry-cured hams were randomly chosen among all the left hams of the trial, to equally represent the two CP contents, the two breeds, and the two sexes, according to a 2 breeds × 2 dietary treatments × 2 sexes factorial design with 5 replications for each combination.

Among the pig breeds used in the feeding trial, we selected for this study the two characterized by the largest differences in term of final back-fat thickness, carcass lean yield and fat cover thickness of the hams^9^, which reflected different aptitudes for lean growth when kept under restricted feeding regime. The “fatter” breed consisted of traditional cross between Italian Duroc boars (D) and Italian Large White (LW) sows selected by the Italian Pig Breeders Association (Anas) according to a breeding programme specifically intended for traditional heavy pig production, with a particular emphasis on ham quality traits^35,36^. The “leaner” breed consisted of pig progeny of commercial Danish Duroc (Danbred) boars mated to crossbred sows of their parent lines.

### Dry-curing processing

After the carcass dissection, raw hams were refrigerated for 24 hours, moved to the ham factory, trimmed and weighed. Fat cover thickness was measured on raw hams with a ruler at the level of the *Biceps femoris* muscle below the femur head of the ham.

At the ham factory, the trimmed hams were salted with sea salt and stored at 2–3 °C for the number of days corresponding to the weight in kg of each fresh ham^37^. After salting, the hams were weighed and salting losses calculated. The hams were then pressed for 48 hours to give hams the typical San Daniele guitar-like shape, and rested for 90 days at 70–80% relative air moisture and 4–6 °C. The hams were then rinsed with cold water, dried for one week, greased with a natural mixture made from lard, salt and cereal meal, ripened in a naturally ventilated room for 15 months, weighed, deboned and weighed again. Seasoning and deboning losses were measured.

### Ham sampling

Just below the femur head the cued hams were cut to obtain three slices of different thicknesses. The first slice (15 mm thick) was used to evaluate water activity and thiobarbituric acid reactive substances (TBARS), reflecting secondary lipid oxidation products. The second slice (14 mm thick) was used for physical and texture analyses, after which the lean part was separated from the fat with a knife, minced and analysed for proximate composition, Na content and FA profile. The separated subcutaneous fat was analysed for FA composition. The third slice (3 mm thick) was ground and analysed for proximate composition of the whole slice including subcutaneous fat.

### Analyses of water activity and secondary lipid oxidation products (TBARS)

The water activity in the lean part of the ham was measured with a dew-point hygrometer (AquaLab 4 TEV, Decagon Devices, Pullman, WA, USA) on squared-shaped samples (15 × 15 mm) taken from the part closest to the *Biceps femoris* muscle.

The lean part and the subcutaneous fat of the slice were separated with a knife and minced, then 2-gram samples of each part were analysed for TBARS^38^. After adding 5 ml of n-heptane and 8 ml of trichloroacetic acid 5%, the samples were homogenized for 30 s at minimum speed using a rod homogenizer (T25 Digital Ultra-Turrax, Ika, Staufen, Germany). Following centrifugation (2834 g for 3 min at 4 °C) the supernatant was removed and 2.5 ml of the lower layer was filtered and mixed with 2.5 ml of thiobarbituric acid (0.02 M) in Pyrex test tubes. The solution was incubated in a Falc SB24 thermostatically-controlled water bath (Falc Instruments, Treviglio, Bergamo, Italy) at 95 °C for 35 min then cooled with running water. The absorbance of the chromatic complex was measured at 532 nm using a UV–Vis spectrophotometer (V-750, Jasco Europe, Cremella, Lecco, Italy) and expressed as milligrams of malondialdehyde/kg of sample using a calibration curve made with solutions of 1,1,3,3-Tetramethoxypropane at scalar concentrations (Sigma-Aldrich, Saint Louis, Missouri, USA).

### Physical and texture analyses

**Figure 5:**
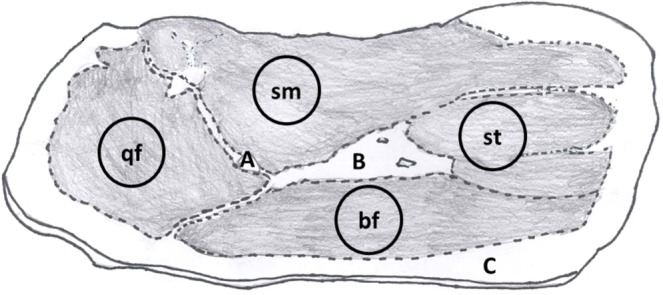
Slice of deboned dry-cured ham. bf: *biceps femoris*; qf: *quadriceps femoris*; sm: *semimembranosus muscle*; st: *semitendinosus muscle*. (**a**) Bone area; (**b**) Fatty area; (**c**) Subcutaneous fat.

Physical and texture analyses were performed on the four muscles of each slice (*Biceps femoris* (Bf), *Quadriceps femoris* (Qf), *Semimembranosus* (Sm), and *Semitendinosus* (St), (Fig. 5). The lightness (L*), green-red (a*) and blue-yellow (b*) components of each muscle were assessed with a Minolta CM-500 colorimeter (Minolta, Osaka, Japan) with 10° standard observer, D65 illuminant and an aperture of 8 mm, according to CIE^39^. The pH was measured on the 4 muscles with a Crison Basic 20 pH meter (Crison SpA, Carpi, Modena, Italy).

The texture profile (TPA) was analysed with a TA.XT plus Texture Analyser (Stable Micro Systems, London, UK) at 15 °C with a 500 N load cell and a 20 mm compression probe. Each of the four muscles was compressed twice to 30% of its original thickness (14 mm) at a speed of 2 mm/s. The Texture Exponent software (Stable Micro System, London, UK) was used to compute the hardness, cohesiveness, adhesiveness, springiness and chewiness of each muscle from the two compression curves, according to Tabilo *et al*.^40^. Hardness (N) was defined as the maximum peak force, which is the force needed to obtain deformation. Cohesiveness (dimensionless) was defined as the ratio between the area under the second curve and the area under the first curve. Adhesiveness (N × s) was the negative area between the two curves, which represents the work needed to overcome the attractive forces between the compression device and the muscle surface. Springiness (dimensionless) was the ratio of the time recorded between the start of the second area and the second probe reversal to the time recorded between the start of the first area and the first probe reversal, which represents the elasticity of the muscle. Chewiness (N) was calculated as hardness × cohesiveness × springiness.

Shear force was measured with a Warner-Bratzler texture analyser (LS5, Ametek Lloyd Instruments, Fareham, UK) equipped with an inverted V-shaped shear blade. Five 1-cm^3^ prisms per muscle were obtained from each sample and cut with a force of 500 N and a speed of 2 mm/s. Shear force was then calculated with the NEXIGEN Plus 3 software (Bognor Regis, UK).

### Chemical analyses

The proximate composition, determined both on the whole slice and on the “lean” part of the slice only (the ham slice without subcutaneous fat), concerned moisture (# 950.46), total protein N × 6.25 (# 981.10), lipids (#991.36) and ash (# 920.153), according to AOAC^41^. The soluble N, determined in trichloroacetic acid 10% solution^42^ and expressed as soluble protein (soluble N × 6.25,), and the proteolysis index, calculated as the percentage ratio between the soluble and total protein, were determined on the lean part of the slice only. Also, Na was determined on the lean part of the slice, using an inductively coupled plasma - optical emissions spectrometer (ICP-OES; Ciros Vision EOP, Spectro Analytical Instruments GmbH, Kleve, Germany) on an aliquot of 1 g of the minced slice, which was mixed with 7 ml of 67% nitric acid and 2 ml of 30% hydrogen peroxide and mineralized at 200 °C for 15–18 min in a microwave digestion system (Milestone Start, Sorisole, Bergamo, Italy). The samples were cooled to 35 °C and made up to volume with distilled water. Salt was calculated as Na × 2.50^43^.

According to Dalla Bona *et al*.^44^ and Schafer^45^, fat was extracted from both the subcutaneous depot (SC) and from the lean part of the slice (intramuscular, IM). IM and SC fat were ground separately and homogenized for 10 s at 4500 g (Grindomix GM200; Retsch, Haan, Düsseldorf, Germany). A sub-sample of 20 to 30 g was stored at −20 °C until analysis. After thawing at ambient temperature, the fat was extracted from a 4.0 g subsample of each part mixed with 15 g of anhydrous sodium sulphate. The mixture was homogenized with a Hydromatrix (Phenomenex, Castel Maggiore, Bologna, Italy) and transferred to 15-mL stainless steel extraction cells for accelerated solvent extraction (ASE, Thermo Fisher Scientific Inc., Waltham, MA, USA) with petroleum ether as the solvent. The extraction conditions were: temperature, 120 °C; pressure, 10 MPa; three static cycles of 1 min each; rinse, 100%; purge, 60 s using 8 mL/sample of fresh solvent^46^. The solvent was evaporated using a rotary film evaporator (Rotavapor® R-205, Buchi Italia s.r.l., Cornaredo, Italy) and samples were placed in an oven at 60 °C for 15 min before being weighed. An aliquot of 40 mg of extracted fat was collected to be methylated according to Christie^47^, with minor modifications. Fat samples were transferred to a test tube fitted with a condenser, to which was added 2 mL of 2% sulphuric acid in methanol.

According to Gallo *et al*.^15^, the mixture was left overnight in a stoppered tube at 50 °C, then 2 ml of n-heptane and water (4 mL) containing potassium bicarbonate (2%) was added. Samples were centrifuged at 2834 g for 10 min, the supernatant was collected with a micropipette and transferred to a vial for gas chromatographic (GC) analysis. The fatty acid (FA) methyl ester contents were determined with an Agilent 7820 GC system (Agilent, Palo Alto, CA, USA) equipped with a flame-ionization detector and an Omegawax 250 capillary column (Omegawax 250, Supelco, Bellefonte, PA, USA; 30 m, 0.25 mm i.d.; film thickness 0.25 μm). The carrier gas was hydrogen at a flow rate of 1 mL min-1.

The GC operative conditions were those described by Dalla Bona^44^. Briefly, a split/splitless injector with a split ratio of 1:80 was used to inject an aliquot of the sample into the GC system under the following conditions: initial oven temperature 60 °C held for 1 min, then increased to 173 °C at a rate of 2 °C/min and held for 30 min, then increased to 185 °C at 1 °C/min and held for 5 min, and finally increased to 220 °C at a rate of 3 °C/min and held for 19 min. The injector temperature was set at 270 °C and the detector temperature at 300 °C. Individual FA methyl esters were identified by comparison with a standard mixture (18918–1AMP 595 N, Supelco, Bellefonte, PA, USA). The FA methyl esters were quantified using methyl 12-tridecenoate as internal standard, and the area of each peak was corrected using flame ionization detector (FID) relative response factors. These response factors were determined using calibrations obtained from five serial dilutions for each standard fatty acid^46^. All calibrations were linear and all R^2^ were > 0.998. The FA composition was expressed as grams per 100 g of total FAs.

### Statistical analysis

Traits with one observation per ham (weights and weight losses during processing, and salt content, soluble protein, proteolysis index, water activity, and TBARS of lean part of the slice) were analysed according to a linear model which included the fixed effects of breed, sex and diet and their interactions.

All the other traits, which presented replications per ham because determined on different muscles (physical and texture traits) or tissues (proximate composition and FA profile), were processed using the SAS MIXED procedure (SAS Inst. Inc., Cary, NC) according to the following linear mixed model:

$$\begin{array}{c}{{\rm{y}}}_{{\rm{ijklm}}}=\mu +{{\rm{BR}}}_{{\rm{i}}}+{{\rm{diet}}}_{{\rm{j}}}+{{\rm{tissue}}}_{{\rm{k}}}+{{\rm{sex}}}_{{\rm{l}}}+{\rm{BR}}\times {{\rm{diet}}}_{{\rm{ij}}}+{\rm{BR}}\times {{\rm{tissue}}}_{{\rm{ik}}}+{\rm{diet}}\times {{\rm{tissue}}}_{{\rm{jk}}}+{\rm{BR}}\times {\rm{diet}}\\ \,\,\,\,\times {{\rm{tissue}}}_{{\rm{ijk}}}+{\rm{ham}}{({\rm{BR}}\times {\rm{diet}}\times {\rm{sex}})}_{m:\text{ijl}}+{{\rm{e}}}_{{\rm{ijklm}}}\end{array}$$

where y_ijklm_ is the observed trait; μ is the overall intercept of the model; BR_i_ is the fixed effect of the ith breed (i = 1, 2); diet_j_ is the fixed effect of the jth feeding treatment (j = 1, 2); tissue_k_ is the fixed effect of the kth muscle (k = 1,…, 4) or part of slice (k = 1, 2); sex_l_ is the fixed effect of the lth gender (l = 1,2; [1 = barrow, 2 = gilt]); BR × diet_ij_ is the effect of the interaction between breed and diet; BR × tissue_ik_ is the effect of the interaction between breed and muscle or part of slice; diet × tissue_jk_ is the effect of the interaction between diet and muscle or part of slice; BR × diet × tissue_ijk_ is the effect of the interactions among breed, diet and muscle or part of slice; ham_m:ijl_ is the random effect of the m^th^ ham (m = 1,…, 40) within BR, diet and sex; e_ijklm_ is the random residual.

Ham within BR, diet and sex, and residuals were assumed to be independently and normally distributed with a mean of zero and variances of σ_ham_^2^ and σ_e_^2^, respectively. In line with the experimental design, the effects of BR, diet, sex and BR × diet were tested using ham within the BR × diet × sex interaction as the error line, whereas the effects of tissue and its interactions were tested on the random residual, according to model used in Schiavon *et al*.^48^.

The 4 degrees of freedom of BR × diet_ij_ interaction were used to test the significance of the differences due to the diet within breed. Similarly, the 4 degrees of freedom of the BR × tissue_ik_ interaction were used to test the significances of the differences due to BR in the chemical compositions of both the whole slice and the lean part of it. Differences among muscles for pH, colour and the texture variables were compared using the Bonferroni correction for multiple testing.
